# Three-dimensional fine structures in deep fascia revealed by combined use of cryo-fixed histochemistry and low-vacuum scanning microscopy

**DOI:** 10.1038/s41598-023-33479-3

**Published:** 2023-04-18

**Authors:** Hiroyuki Imazato, Nobuyasu Takahashi, Yusuke Hirakawa, Yoichiro Yamaguchi, Masaru Hiyoshi, Takuya Tajima, Etsuo Chosa, Akira Sawaguchi

**Affiliations:** 1 Division of Orthopaedic Surgery, Faculty of Medicine, University of Miyazaki, 5200 Kihara, Kiyotake, Miyazaki 889-1692 Japan; 2 Department of Anatomy, Ultrastructural Cell Biology, Faculty of Medicine, University of Miyazaki, Miyazaki, 889-1692 Japan

**Keywords:** Anatomy, Medical research

## Abstract

Recent physiological studies have shown that the deep fascia has received much attention concerning clinical medicine; however, histological examination of the deep fascia has not been well established. In this study, we aimed to clarify and visualize the structure of the deep fascia by taking advantage of cryofixation techniques and low-vacuum scanning electron microscopy. As a result, the ultrastructural observations revealed three-dimensional stratification of the deep fascia composed of three layers: the first superficial layer consisting of collagen fibers extending in various directions with blood vessels and peripheral nerves; the second intermediate layer formed by single straight and thick collagen fibers with flexibility; and the third deepest layer, consisting of relatively straight and thin collagen fibers. We explored the use of two hooks to hold a piece of deep fascia in place through the course of cryo-fixation. A comparative observation with or without the hook-holding procedure would indicate the morphological adaptation to physiological stretch and contraction of the deep fascia. The present morphological approach paves the way to visualize three-dimensional ultrastructures for future biomedical studies including clinical pathophysiology.

## Introduction

For many years, the deep fascia has been recognized as a structure that simply envelops muscles^1,2^; however, recent studies have revealed that the deep fascia has multiple functions^3,4^. Gallegos et al.^3^ indicated that the fascia is important for wound healing, and Taguchi et al.^4^ demonstrated that the deep fascia is important not only for nociception but also as a target tissue for treatment in patients with myofascial pain. Myofascial trigger point injection therapy was popularized in the 1950s^5^ and is still used worldwide for the treatment of medical conditions^6,7^. In addition, deep fascia can be treated and operated on in daily orthopedic surgery, rehabilitation, and reconstructive surgery^8–10^. Myofascial release has also received significant attention in sports medicine as a treatment of deep fascia^11,12^. Despite the growing interest in fascia^13^, there is still a lack of comprehensive anatomical and histological descriptions of this structure. Therefore, the importance and demand for an accurate anatomical structure of the deep fascia have been increasing.

Histological examination of the deep fascia is currently not well established. Although several studies have addressed the histological characteristics of the deep fascia^14,15^, the microstructure, muscle protection, blood flow, and innervation have not yet been clarified in detail. As a result, the inconsistent use of the anatomical term 'fascia' makes it difficult to compare the results across research studies and to draw more generalized conclusions in a clinical study^2,16^.

Conventional methods of analyzing morphology are limited in that they have the possibility of producing artifacts during sample preparation. The use of chemicals has been demonstrated to produce variable effects on specimens, including shrinking, dehydration, swelling, and rehydration, as well as alterations in mass, coloration, texture, and chemical composition^17^. The predominant approach for histological studies is a fixation with 4% paraformaldehyde (PFA) and formalin; however, chemical fixation protocols have been shown to play a crucial role in the introduction of artifacts^18^. The formalin-fixed muscle tissue was found to have decreased mass and density^19^. Also, the fixation significantly decreased fiber area in the deep fascia with a strong correlation^18^. Chemical fixation may be a suitable method for simple and preliminary observations but not for analyzing the layered structure of the deep fascia.

Cryo-immobilized processing is widely recognized as superior to chemical fixation in the preservation of adequate morphological structures, close to the living state^20–23^. Plunge freezing in a cooled propane/isopentane mixture has been effectively employed in immunohistochemical studies for light microscopy^20^. Nonetheless, this technique can only be applied to relatively small and thin tissue within 300–400 μm of the contact surface of a cooled liquid isopentane/propane mixture^23^. To adequately assess the structure of the deep fascia, suitable sample fixation for deep fascial cross-sections under the same conditions as in vivo are essential.

Recently, we reported the utility of low-vacuum scanning electron microscopy (LVSEM)^24^. This allows for a simple three-dimensional (3D) cell/tissue architecture survey method where the sample is embedded in the same paraffin sections as those used for histological analysis, such as Hematoxylin–Eosin (HE) staining. In general, SEM provides 3D information about specimen surfaces by collecting backscattered electrons (BSE) reflected from the surface, as well as secondary electrons (SE) which are forced out of the surface^25–28^. While conventional SEM is capable of imaging the 3D microstructure of samples with high resolution, it is unsuitable for non-conductive paraffin sections because of the negative charge accumulation. LVSEM allows for BSE and/or SE imaging of non-conductive paraffin samples by eliminating negative charge accumulation using positive ions within residual gas molecules^29,30^. Paraffin wax is inexpensive and easily handled for sectioning for LVSEM; in contrast, conventional SEM sample preparation requires skill and is time-consuming^24^. Moreover, LVSEM enables retrospective investigation of vintage paraffin samples, which can be observed in 3D and high magnification^31^.

This study aimed to clarify and visualize the fine structures within deep fascia using various histological and cryofixation techniques^20–23^ and LVSEM. This novel combined approach allows us to better analyze fine structures in 3D and can provide new insights into the structure of the deep fascia.

## Results

### Schematic illustration

**Figure 1:**
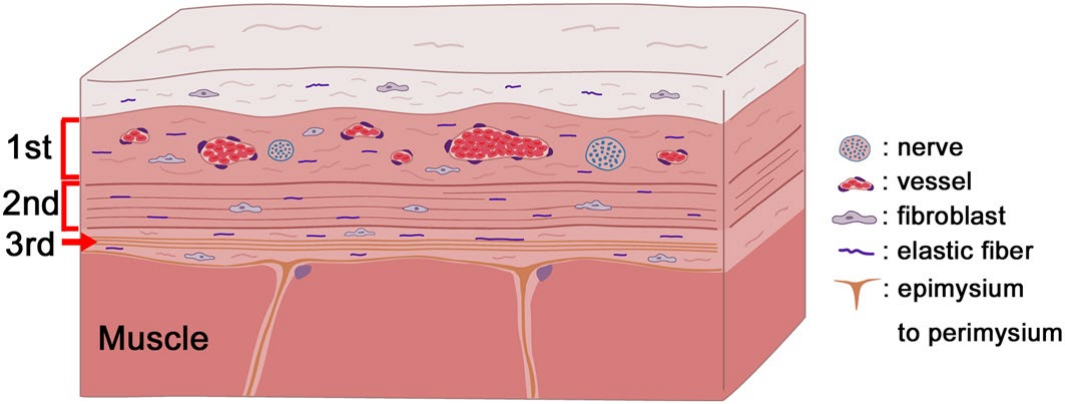
Schematic representation of the deep fascia, which we propose to be divided into three layers: the first superficial layer, containing collagen fibers extending in various directions with abundant vessels and nerves; the second intermediate layer, composed of single straight and thick collagen fibers with fibroblasts; and the third deepest layer, composed of straight and thin collagen fibers. A thin epimysium exists underneath the deep fascia and is connected to the perimysium. Loose connective tissue is identified between the second and third layers. Elastic fibers are found in all layers.

We propose that the deep fascia can be divided into three layers. A schematic illustration of the deep fascia is shown in Fig. 1. The first superficial layer is loose and is formed of collagen fibers extending in various directions with abundant vessels and nerves. The second intermediate layer is composed of single straight and thick collagen fibers with fibroblasts. It may contribute to changes in the structure as the surrounding environment is stretched and contracted. Several blood vessels and nerves are present across the first and second layers. The third and deepest layer consists of relatively straight and thin collagen fibers with many elastic fibers. A thin epimysium also exists beneath the deep fascia and is connected to the perimysium. Many elastic fibers are identified in all layers of the deep fascia.

### Conventional chemical (PFA) fixation

**Figure 2:**
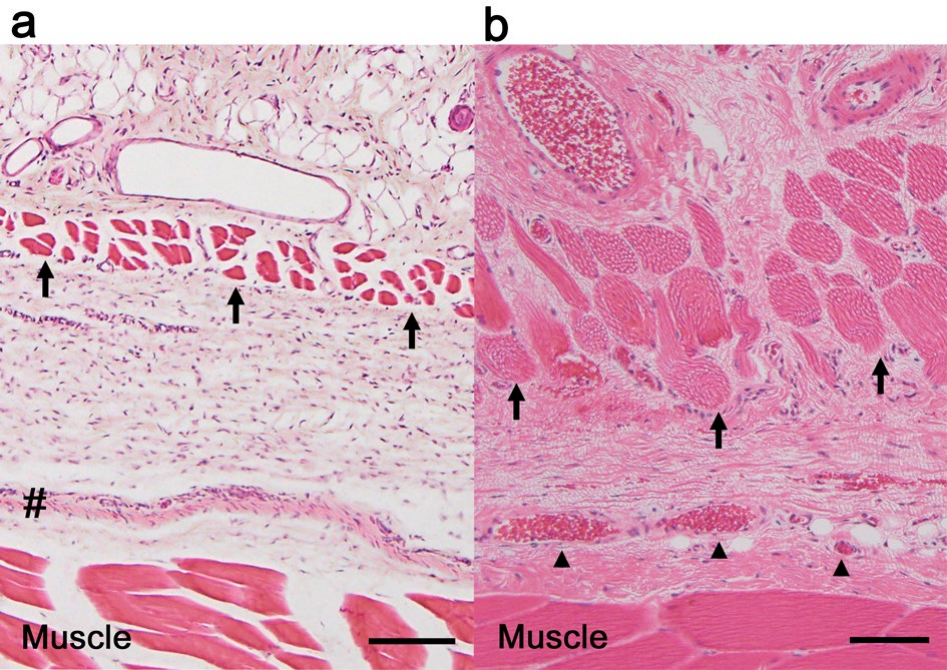
Micrographs of the sequential section of skin and rectus femoris muscle stained with hematoxylin–eosin (HE). (**a**) Conventional fixation. In general, the rat has a ‘panniculus muscle (arrow)’^33^ between the subcutaneous tissue and the deep fascia. Contraction of the panniculus and rectus muscles resulted in decreased volume, and a strongly contracted band (#) was observed in the deep fascia. Scale bar = 100 µm. (**b**) Plunge freezing and freeze-substitution. The panniculus muscle (arrow) and the rectus femoris muscle were thick and not contracted. The presence of blood vessels (arrowhead) within the deep fascia is clearly shown, and the strongly contracted band is not identified. The deep fascia is still not well-structured. Scale bar = 100 µm.

The sequential HE staining section of the skin and rectus femoris muscle revealed dissociation around the deep fascia and muscles (Fig. 2a). Part of the deep fascia strongly contracted, and the rectus femoris and panniculus muscle decreased in volume. A strongly contracted band in the deep fascia was noted. Additionally, the tissue surrounding the contracted deep fascia was sparse and displayed less staining. Chemical fixation of the specimen may disrupt the connection around the deep fascia, making it inadequate for observing the fine structure.

### Plunge freezing followed by freeze-substitution

Figure 2b shows the sequential HE staining section of the same area (Fig. 2a) fixed by plunge freezing followed by freeze-substitution. The deep fascia was thicker than conventional fixation without contraction. The strongly contracted band was absent, and blood vessels containing abundant erythrocytes within the deep fascia were clearly shown. Moreover, the rectus femoris and panniculus muscles were not contracted. The presence of blood vessels (arrowhead) within the deep fascia was clearly shown. Nevertheless, the structure of the deep fascia still contained flaws.

**Figure 3:**
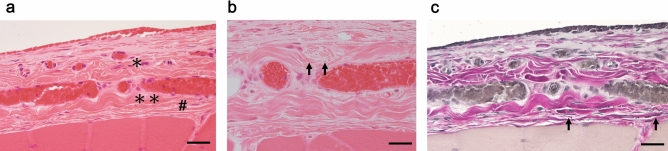
Micrographs of the exposed deep fascia without hook-holding used in plunge freezing followed by freeze-substitution technique. (**a**) Hematoxylin–eosin (HE) stain. The deep fascia has abundant vessels, particularly, the first superficial layer (*) with collagen fibers extending in various directions. The second intermediate layer was formed by strongly wavy and thick bundles of collagen fibers (**). The third, deepest layer displayed straight and thin bundles of collagen fibers (#). Scale bar = 20 µm. (**b**) HE stain. The nerve tissue was identified (arrow) in the first superficial layer. Scale bar = 15 µm. (**c**) Elastica Van Gieson (EVG) stain. Collagen bands were forming clearly different layers. Elastic fibers (arrow) were identified in all layers of the deep fascia. Scale bar = 20 µm.

**Figure 4:**
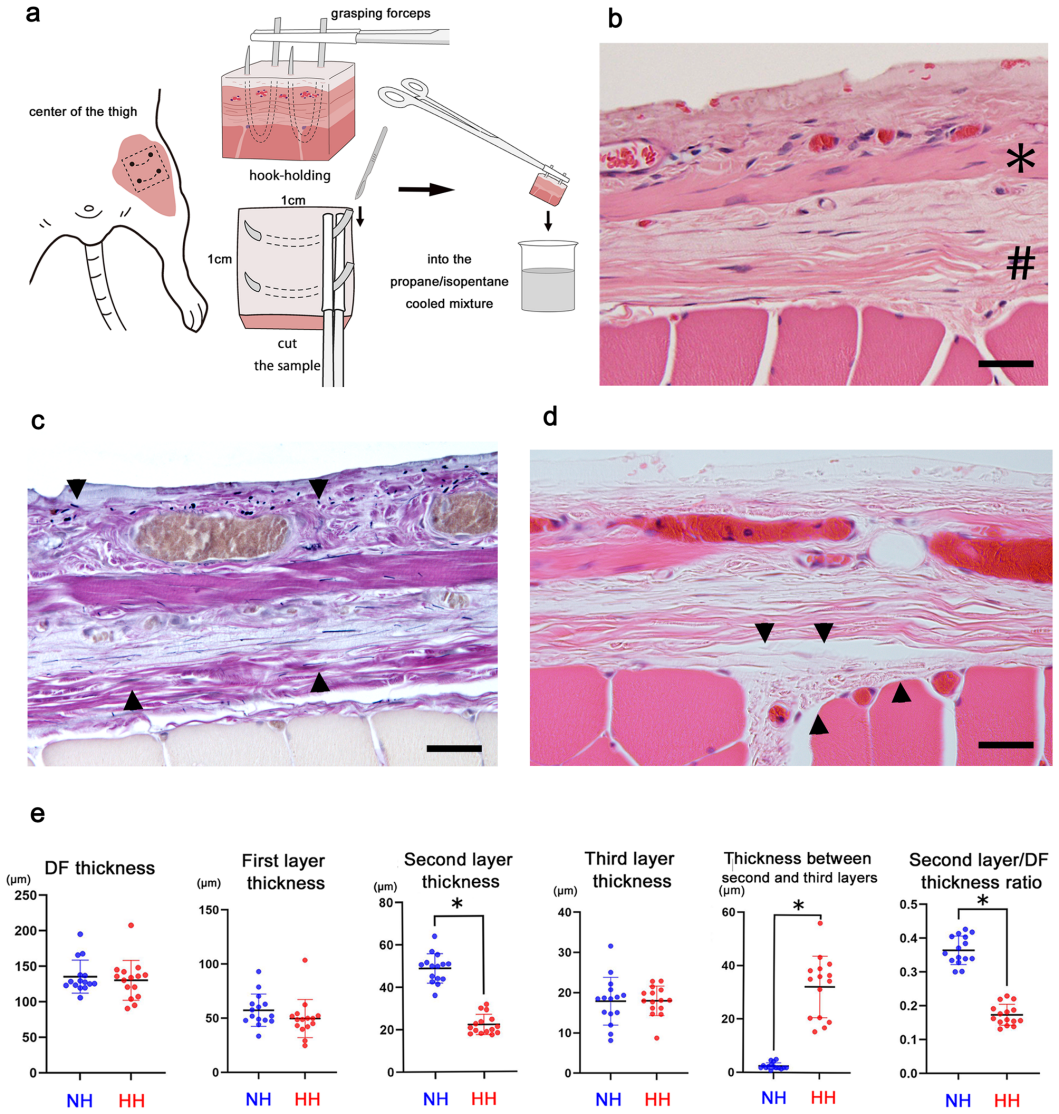
Plunge freezing followed by freeze-substitution with the hook-holding procedure. (**a**) Scheme illustrating the hook-holding procedure. To retain the morphology, we held the deep fascia and rectus femoris muscle with two hooks and grasping forceps, then excised it at 1 cm^2^ and plunged it into the cooled propane/isopentane mixture. Subsequently, plunge freezing followed by freeze-substitution was carried out. (**b**) Hematoxylin–eosin (HE) stain. In the first superficial layer, collagen fibers extend in various directions along with vessels replete with erythrocytes. The second intermediate layer (*) contains single straight and thick collagen fibers. The third deepest layer (#) is composed of straight and thin collagen fibers. Lightly stained loose connective tissue is observed between the second and third layers. Scale bar = 50 µm. (**c**) Elastika Van Gieson stain. Collagen bundles in the second intermediate layer are almost straight. Elastic fibers are observed in all layers of the deep fascia (arrowhead). Scale bar = 50 µm. (**d**) HE stain. The epimysium may exist independently of the third layer of the deep fascia and connected to the perimysium (arrowhead). Scale bar = 50 µm. (**e**) A quantitative comparison of the mean thicknesses of the deep fascia, first, second, and third layers, between the second and third layers, and the thickness ratio of the second layer to the deep fascia was conducted for 15 subjects per group. All data are presented as the mean ± standard deviation (SD). Statistical significance is determined using a Student’s *t*-test, with asterisks indicating statistically significant differences (*p < 0.05). NH, no hooks; HH, Hook-holding procedure; DF, deep fascia.

Figure 3 demonstrates plunge freezing followed by the freeze-substitution technique. Plunge freezing only takes effect within 300–400 μm of the contact surface of the liquid nitrogen^23^; therefore, the skin was excised to expose the deep fascia and rectus femoris muscle. The first superficial layer was formed by collagen fibers extending in various directions, with blood vessels containing abundant erythrocytes (Fig. 3a) and peripheral nerves (Fig. 3b). Compared with Fig. 4b, the second intermediate layer was formed by strongly wavy collagen bundles, while the collagen fibers in the third deepest layer were straight and thin. The loose connective tissue between the second and third layers was not identified. Elastic fibers stained with EVG were observed in all layers (Fig. 3c). These findings may indicate that the deep fascia shrunk when the specimen was cut; therefore, the hook-holding procedure may be necessary for preserving the structure of the deep fascia in vivo.

### Hook-holding procedure with plunge freezing followed by freeze-substitution

A target portion of the sample that includes the rectus femoris muscle was identified. Two hooks were then placed at a distance of 8 mm to prevent a contraction during sample preparation (Fig. 4a). Deep fascias are composed of three layers without prominent artifacts and dissociation of structures identified in Fig. 4b. The first superficial layer was formed by collagen fibers extending in various directions with vessels containing abundant erythrocytes. The second intermediate layer was formed of single straight and thick collagen fibers. Elastic fibers were identified in all layers via Elastica Van Gieson (EVG) staining (Fig. 4c). Lightly stained loose connective tissue was observed between the second and third layers (Fig. 4b). Thin collagen fibers ran straight in the third layer (Fig. 4c, d). The epimysium was different from the thin multilayered collagen layers and connected to the perimysium (Fig. 4d). Together with these findings, we indicated that the deep fascia consists of three different layers and is an independent structure from the epimysium (Fig. 1).

Further, we compared the hook-holding procedure with other options (Table 1). The thickness of the deep fascia, as well as the first and third layers, did not differ from those obtained without the hook-holding procedure (Fig. 4e). However, the thickness of the second layer was significantly lower than that obtained without the hook-holding procedure (p < 0.05). Similarly, the thickness ratio of the second layer to the deep fascia was significantly lower than that obtained without the hook-holding procedure (p < 0.05). These results showed that overall, the thickness of the deep fascia remained unchanged, but significant morphological changes were observed in the second layer. In contrast to the second layer (which decreased in thickness), the thickness of the loose connective tissue between the second and third layers was significantly increased (p < 0.05). This result would probably cause the morphological changes of the second layer by waving and running straight in conjunction with the surrounding environment for constancy.

**Table 1:** Quantitative comparison of the thickness of the deep fascia, each layer, loose connective tissue between the second and third layers, and second layer/deep fascia thickness ratio with or without the hook-holding procedure.

Thickness (μm)	No hooks Mean ± SD	Hook-holding procedure Mean ± SD	Statistical analysis
Deep fascia	135.3 ± 22.4	130.3 ± 27.1	n.s.
First layer	57.3 ± 14.4	49.7 ± 17.1	n.s.
Second layer	48.4 ± 6.7	22.2 ± 4.6	p < 0.05
Third layer	17.9 ± 5.7	18.0 ± 3.5	n.s.
Between the second and third layers	2.41 ± 1.2	32.2 ± 11.1	p < 0.05
Second/deep fascia layer ratio	0.36	0.17	p < 0.05

SD, standard deviation; n.s., not significant.

### Low-vacuum scanning electron microscopy

**Figure 5:**
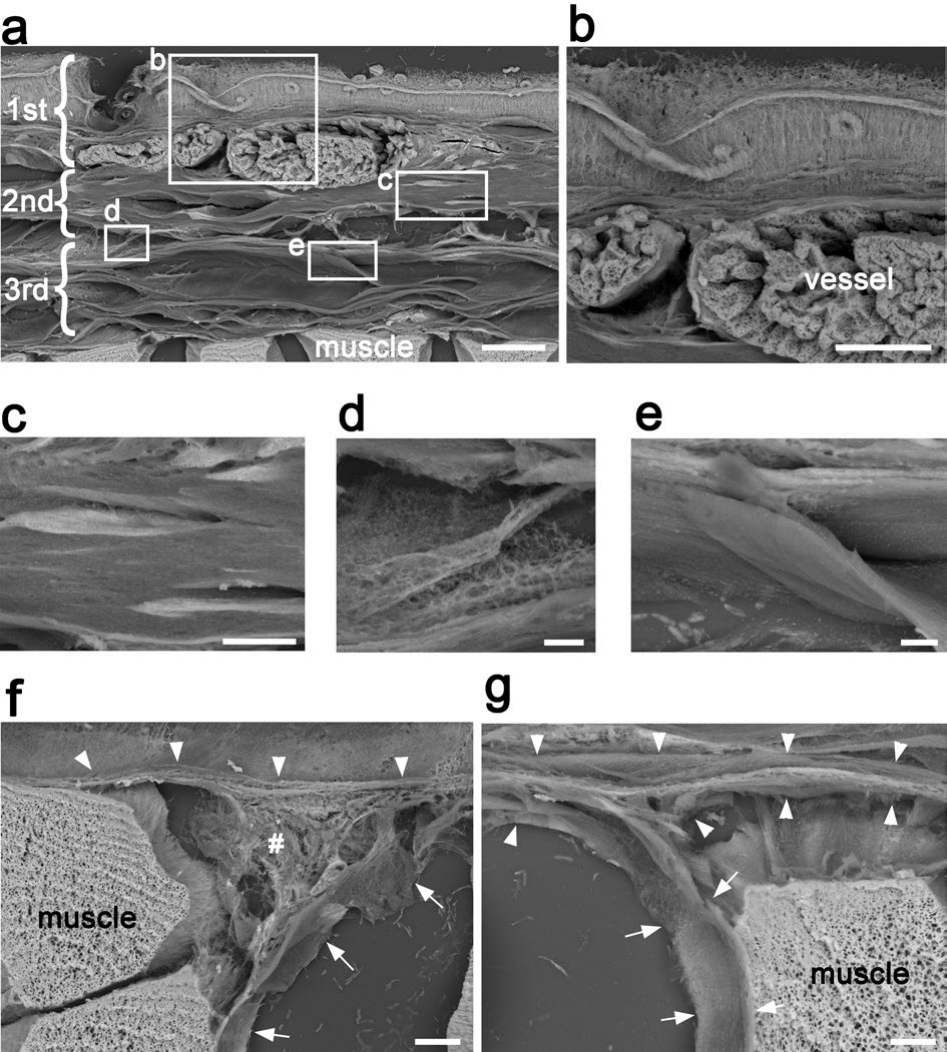
LVSEM of 20-µm sections with hook-holding procedure. (**a**) Image of the deep fascia and rectus femoris muscle of the thick paraffin section from the same block used in Fig. 4b. The deep fascia is separated into three layers from a 3D perspective. Squares in this figure are magnifications of these areas. Scale bar = 30 µm. (**b**) The first superficial layer of the deep fascia is composed of collagen fibers extending in various directions with round vessels repleting abundant erythrocytes. Scale bar = 10 µm. (**c**) The second intermediate layer underneath the first superficial layer is formed of straight and thick bundle. Scale bar = 5 µm. (**d**) The loose connective tissue is identified between the second and the third layer. Extensive networks of extremely thin fibers are noted. Scale bar = 3 µm. (**e**) The third deepest layer is formed by straight and thin collagen fibers. Slight undulation of the surface of the collagen fiber is observed. Scale bar = 3 µm. (**f** and **g**) The epimysium (arrowhead) is different from the third deepest layer and clearly connects to the perimysium (arrow). Loose connective tissue (#) is identified among the epimysium, the perimysium and the muscle. Scale bar = 10 µm.

The LVSEM procedure enabled us to survey a whole section on a centimeter scale (Fig. 5a), in contrast to the millimeter-scale sample size of conventional electron microscopy^24^. We observed the samples of the deep fascia and rectus femoris muscle in Fig. 4b using LVSEM. The overall view of the deep fascia using LVSEM indicated that it was composed of three different characteristics. The first superficial layer of the deep fascia was composed of collagen fibers extending in various directions, along with vessels (Fig. 5b). The second intermediate layer formed single straight and thick bundle (Fig. 5c). The loose connective tissue was identified between the second and third layers (Fig. 5d). The third deepest layer was formed by straight and thin collagen fibers. A slight undulation of the surface of the collagen fiber was observed (Fig. 5e). The epimysium existed under the third deepest fascia and was connected to the perimysium (Fig. 5f and g). The connection between the epimysium and the perimysium may indicate that the epimysium is independent of the deep fascia. These findings are consistent with those of light microscopy.

### Transmission electron microscopy

**Figure 6:**
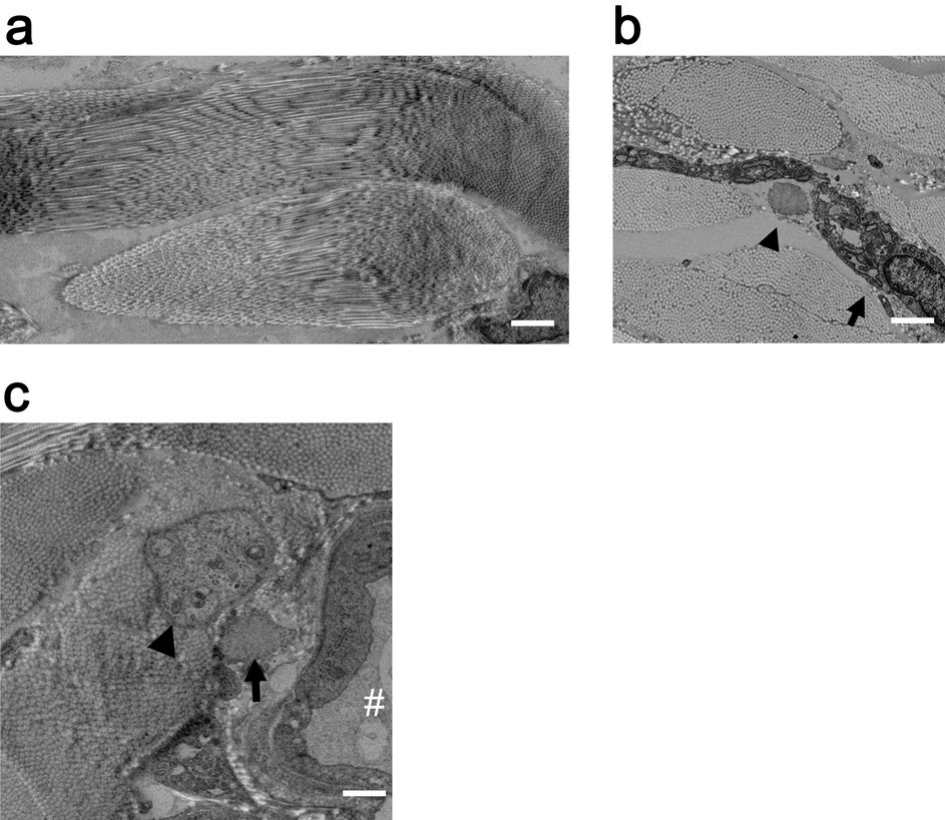
TEM of the deep fascia. (**a**) Bundles of collagen fibers in the second intermediate layer displayed wavy structures heading in different directions. Scale bar = 3 µm. (**b**) Cut surface of elastic fiber (arrowhead) was observed nearby the fibroblast (arrow). Scale bar = 1 µm. (**c**) Cut surface of the axon of the unmyelinated nerve (arrowhead) was noted near the vessel (#). Elastic fiber (arrow). Scale bar = 3 µm.

Many collagen fibers forming bundles were positioned in different directions (Fig. 6a). Among them, elastic fibers and fibroblasts were present (Fig. 6b). The axon of the unmyelinated nerve and elastic fiber near the blood vessel were surrounded by bundles of collagen fibers (Fig. 6c).

## Discussion

We employed cryofixed histochemistry, LVSEM, and a morphological approach with a hook-holding procedure to reveal the detailed structure of the deep fascia. To the best of our knowledge, this study is the first to closely observe the fine microstructure of the deep fascia in a state as close to living as possible. Our figures and findings, which have not been previously shown in histological studies, provide novel insights into the microstructure of the deep fascia and indicate that the deep fascia is composed of three layers with distinct characteristics.

Since the microstructure of the deep fascia is so difficult to study, it has not been extensively researched in the past^2^. Some studies have reported that the deep fascia was hypercontracted and collapsed, even in human specimens^14,16^. Furthermore, the relationship between the shallow subcutaneous tissue and the muscle was separated from each other, which is similar to our findings for conventional chemical fixation. Although the importance of the deep fascia has been gradually clarified in recent years^13^, its microstructure and histological characteristics are still unknown^3^. This study is the first to demonstrate the fine microstructure of the deep fascia. Using cryofixation, we identified that the deep fascia contained abundant vessels and nerves in the first superficial layer. These findings presumably indicate the role of fascia as a blood supply to the skin^32^ and the possible origin of pain.

In a previous histological study, Blasi et al.^14^ and Stecco et al.^16^ reported on human deep fascia of the trunk and thighs. These studies demonstrated a variety of figures, ranging from the deep fascia to the muscle; however, the deep fascia was excessively contracted, far from the original structure. Stecco et al.^16^ reported that the deep fascia was divided into two components: the deep layer, a thin layer that displayed the same characteristics as an epimysium and was formed by collagen fibers and many elastic fibers; and the median layer, composed of three layers of parallel collagen fiber bundles. Although they demonstrated a schematic diagram of the median layer as different sheets running horizontally, vertically, and diagonally^16^, the histological figures were ambiguous. Because such layered structures running in different directions were not observed in our study, we questioned the authenticity of the layered schematic diagram.

The deep fascia has flexibility and loose connection to subcutaneous tissue and muscle^1^. Conventional chemical fixation may have prevented the visualization of this flexible structure. Cryofixation is superior to chemical fixation in preserving adequate morphological structures close to their living state^20–23^. The deep fascia is susceptible to chemical fixation (e.g. formalin). Such fixation correlates with a shrinkage of the deep fascia and the muscle tissue, inducing a significant reduction of fiber area^17,19^. To analyze the deep fascia as a living state, we performed a plunge freezing procedure and freeze substitution^20–23^. Plunge freezing in cooled liquid isopentane/propane mixture can only take effect within 300–400 μm of the contact surface^23^, thus we excised the skin of the sample and exposed the deep fascia. Our method revealed the fine structure of the deep fascia, however, the second intermediated layer showed a strongly wavy structure. As a result of cutting the specimen, the deep fascia shrunk. We hypothesized that the deep fascia formed a straight bundle and had flexibility. Therefore, we demonstrated the hook-holding procedure to maintain the original length in vivo. Comparing samples with and without the hook-holding procedure verified the morphological adaptation of the deep fascia to physiological stretch and contraction. Although the overall thickness of the deep fascia remained unchanged, the second layer displayed significant morphological changes. The thick collagen bundles in the second layer may become wavy or linear when the surrounding environment is stretched or contracted.

LVSEM was suitable for electron microscopic observation of a whole-section survey at the centimeter scale without negative charge accumulation on the non-conductive paraffin sections^24^. Thick paraffin sections enabled us to view conventional sections using 3D imaging rather than light microscopy. Our study clearly shows the 3D architecture of the round vessels with abundant erythrocytes in the first superficial layer extending in various directions, and the straight and thick collagen bundle in the second layer. These findings were consistent with those noted in the section subjected to light microscopy from the same paraffin block. LVSEM revealed the ultrastructure of the clear loose connective tissue that was difficult to observe by light microscopy. Moreover, straight thin collagen bundles in the third deepest layer, and the connection between the epimysium and the perimysium, were identified. LVSEM can be used without special techniques to observe the microstructure of the sections from the same paraffin block, leading to new insights concerning morphology.

In conventional chemical fixation, the collagen bundles of the deep fascia were excessively contracted, indicating that the inflexible deep fascia could affect the internal nerves and cause pain. These findings may assist clinicians in the performance of myofascial release by helping them visualize the precise location of trigger point injections. Together with these findings, the deep fascia may function as an ‘organ’, rather than just being a simple membrane.

One limitation of the study is that we only reported on the deep fascia of the thigh. Furthermore, the panniculus muscle, which is unique to rats, does not exist in humans^33^. Freeze-substitution using t-butyl alcohol for avoiding destruction due to surface tension in conventional SEM may generate artifacts. Similarly, the air drying of conventional paraffin sections used in LVSEM may lead to artifacts. Future studies should be aimed at observing and evaluating human deep fascia as well as the whole body. We did not observe the deep fascia under maximum muscle extension or contraction, which may help clarify its function and should be a subject for future study.

In conclusion, we found that the deep fascia is composed of three layers: the first superficial layer was composed of vessels and nerves with collagen fibers extending in various directions; the second intermediate layer was formed by single straight and thick collagen fibers and may be capable of changing form, inducing flexibility against extension and contraction; and the third deepest layer was consisting of straight and thin collagen fibers, which differs from the epimysium that connects to the perimysium. Further application of these techniques and structural findings to clinical experiments including functional analysis may be required to elucidate deep fascia-associated disorders.

## Methods

### Sample preparation

Male Wistar rats (10 weeks) (Kyudo, Kumamoto, Japan) were used in this study. Three to four rats were used in each experimental group. Deep anesthesia was performed via isoflurane inhalation. All animal procedures were carried out according to protocols approved by the University of Miyazaki Animal Research Committee (Approval number: 2019-513-2), and all experiments were performed according to the institutional guidelines of the Animal Experiment Committee. The Animal Research: Reporting of In Vivo Experiments (ARRIVE) essential guidelines were used to formulate the study design, sample preparation, result observation, and data analysis procedures.

### Conventional chemical (PFA) fixation

After deep anesthesia, 100 mL of phosphate-buffered saline at 40 ℃ was perfused through the left ventricle at 3 mL per second by drip infusion, after making a small incision in the right atrium to remove the blood. Next, rats were perfused with 4% PFA in 0.1 M phosphate buffer (pH 7.4) from the left ventricle of the heart by the same velocity and appliance. The hip joint was maintained at 0 degrees of extension and the knee joint at 0 degrees of extension. The front of the thigh was excised from the skin to the rectus femoris muscle by 1 cm^2^ and fixed by immersion in fixative for 2 h at room temperature (RT). After washing in running tap water for 2 h, the samples were dehydrated in a graded series of ethanol, cleared with xylene for 2 h using an automatic tissue processor (TP 1020; Leica Microsystems, Wetzlar, Germany), and embedded in paraffin (melting point 54–56 °C; Wako Pure Chemical Industries, Osaka, Japan) using a heated paraffin embedding station (HistoCore Arcadia H, Leica Microsystems GmbH).

### Plunge freezing followed by freeze-substitution

Plunge freezing followed by freeze-substitution was carried out for light microscopy as previously described, with minor modifications^20–23^. After deep anesthesia, small pieces of sequential sections from the skin to the rectus femoris muscle and the deep fascia to the rectus femoris muscle were excised by 1 cm^2^ and plunged into 30–40 mL of a liquid isopentane/propane mixture cooled using liquid nitrogen (LN_2_). After immersion for at least 20 s, the samples were quickly transferred into LN_2_ and held there until further processing with the freeze-substitution. Freeze substitution was carried out in 0.1% glutaraldehyde in acetone at − 80 ℃ for 16 h and then gradually warmed (− 50 ℃ for 2 h, − 20 ℃ for 1 h, and 4 ℃ for 1 h) to RT. After washing with pure ethanol, specimens were embedded in paraffin.

### Hook-holding procedure with plunge freezing followed by freeze-substitution

After deep anesthesia, a longitudinal skin incision was performed anterior to the center of the thigh to expose the deep fascia. The deep fascia and rectus femoris muscle were held with two hooks (22 mm, 1/2 circle, blunt point) and fixed using grasping forceps to maintain the original length and structure of the deep fascia (Fig. 2a) in vivo. The 1 cm^2^ specimen around the two hooks was carefully excised, then plunged into 30–40 mL of a liquid isopentane/propane mixture cooled by LN_2_. Next, we performed the same process of plunge freezing followed by freeze-substitution, as described above.

### Histological analysis

Paraffin sections (5 µm thick) obtained using the conventional chemical method (4% PFA fixation) and plunge freezing followed by freeze substitution with or without the hook-holding procedure were deparaffinized, rehydrated, and stained with hematoxylin and eosin (HE) for morphological examination. EVG staining was performed according to the manufacturer’s instructions. Axial planes of the rectus femoral muscle were used for all sections.

To verify the morphological changes, a comparative histological analysis of two groups was performed: with and without the hook-holding procedure. The thicknesses of the deep fascia, first layer, second layer, third layer, and between the second and third layers were measured using imaging software (cellSens; Olympus, Tokyo, Japan). We assessed the average thickness of the five samples of two groups on three different cross-section lengths (center and each end of the field) in a high-power field and calculated the mean ± standard deviation. Statistical comparisons were conducted for the two groups.

### Low-vacuum scanning electron microscopy

Paraffin sections (20-µm thick) obtained after plunge freezing and freeze substitution with two needles were deparaffinized in xylene, rehydrated, stained with 1.0% uranyl acetate in 70% methanol for 5 min, washed with distilled water, and stained with Reynolds’ lead citrate solution for 3 min. After washing with distilled water, the specimens were dried at room temperature for 2 h. The microscope slides were placed on the wide stage of the specimen holder using adhesive conductive tape and then placed in an LVSEM (TM4000Plus; Hitachi High-Tech, Tokyo, Japan) operating at 15 kV. Topographic images were reconstructed using Hitachi map 3D software (Hitachi High-Tech).

### Transmission electron microscopy

For morphological observation with transmission electron microscopy **(**TEM), small pieces of the deep fascia were immersed in a mixture of 2% PFA, 2.5% glutaraldehyde, and 0.1 M phosphate buffer (PB; pH 7.4). After rinsing with 0.1 M PB, they were post-fixed with 1% osmium tetroxide in 0.1 M PB, dehydrated in a graded series of ethanol, and embedded in epoxy resin. Ultrathin sections (60–80 nm thick) were cut and stained with uranyl acetate and lead citrate for observation via TEM (HT7700; Hitachi High-Tech).

### Statistical analysis

All data are expressed as the mean ± standard deviation. Statistical significance was assessed using the Student’s *t*-test. All analyses were performed with JMP Pro version 16 (SAS Institute Inc., Cary, NC, USA). Statistical significance was set at p < 0.05.

## Data Availability

All figures and any other information are available upon request from the corresponding author.
